# Heme Oxygenase-1: An Anti-Inflammatory Effector in Cardiovascular, Lung, and Related Metabolic Disorders

**DOI:** 10.3390/antiox11030555

**Published:** 2022-03-15

**Authors:** Stefan W. Ryter

**Affiliations:** Proterris, Inc., Boston, MA 02118, USA; stefan.ryter@proterris.com

**Keywords:** cardiovascular disease, bilirubin, carbon monoxide, heme oxygenase, inflammation, kidney disease, lung disease, metabolic disease

## Abstract

The heme oxygenase (HO) enzyme system catabolizes heme to carbon monoxide (CO), ferrous iron, and biliverdin-IXα (BV), which is reduced to bilirubin-IXα (BR) by biliverdin reductase (BVR). HO activity is represented by two distinct isozymes, the inducible form, HO-1, and a constitutive form, HO-2, encoded by distinct genes (*HMOX1*, *HMOX2*, respectively). HO-1 responds to transcriptional activation in response to a wide variety of chemical and physical stimuli, including its natural substrate heme, oxidants, and phytochemical antioxidants. The expression of HO-1 is regulated by NF-E2-related factor-2 and counter-regulated by Bach-1, in a heme-sensitive manner. Additionally, *HMOX1* promoter polymorphisms have been associated with human disease. The induction of HO-1 can confer protection in inflammatory conditions through removal of heme, a pro-oxidant and potential catalyst of lipid peroxidation, whereas iron released from HO activity may trigger ferritin synthesis or ferroptosis. The production of heme-derived reaction products (i.e., BV, BR) may contribute to HO-dependent cytoprotection via antioxidant and immunomodulatory effects. Additionally, BVR and BR have newly recognized roles in lipid regulation. CO may alter mitochondrial function leading to modulation of downstream signaling pathways that culminate in anti-apoptotic, anti-inflammatory, anti-proliferative and immunomodulatory effects. This review will present evidence for beneficial effects of HO-1 and its reaction products in human diseases, including cardiovascular disease (CVD), metabolic conditions, including diabetes and obesity, as well as acute and chronic diseases of the liver, kidney, or lung. Strategies targeting the HO-1 pathway, including genetic or chemical modulation of HO-1 expression, or application of BR, CO gas, or CO donor compounds show therapeutic potential in inflammatory conditions, including organ ischemia/reperfusion injury. Evidence from human studies indicate that HO-1 expression may represent a biomarker of oxidative stress in various clinical conditions, while increases in serum BR levels have been correlated inversely to risk of CVD and metabolic disease. Ongoing human clinical trials investigate the potential of CO as a therapeutic in human disease.

## 1. Introduction

Heme oxygenase-1 (HO-1), a stress protein and metabolic enzyme, continues to attract worldwide basic and translational research interest as a regulator of cellular and tissue homeostatic responses, a modulator of immune function and host defense, and mitigator of inflammation [1,2,3,4,5,6]. HO-1, the major inducible isozyme, and its constitutively regulated isozyme, HO-2, convert heme to three catabolic products: biliverdin-IXα (BV), ferrous iron (Fe^2+^), and carbon monoxide (CO) [7]. BV generated by HO activity is subsequently converted to BR by the action of NAD(P)H: biliverdin reductase (BVR) [8]. The recognized anti-inflammatory, cytoprotective, and immunomodulatory effects of HO-1, and its enzymatic reaction products, are now known to collectively have broad significance in human disease pathogenesis [5,6,9,10,11]. According to its primary catabolic activity, HO-1 reduces the bioavailability of free heme and thereby confers an important anti-oxidative and anti-inflammatory function in disease [12,13]. Heme is a recognized pro-inflammatory mediator that may trigger pathological responses, including stimulation of Toll-like receptor 4-(TLR4) dependent inflammatory responses, membrane lipid peroxidation, and ferroptosis [14,15,16,17]. The biological activities of HO-dependent reaction products including CO, Fe^2+^, and BR (derived from BV) may contribute individually or in concert to the known cytoprotective properties of HO-1 [9,18]. Of these, BR has gained prominence as a physiological antioxidant and inverse risk factor for cardiovascular disease (CVD) and metabolic diseases [19,20,21], and has also recently gained recognition as a paracrine metabolic regulator [22]. CO, released from HO activity, is now recognized as an endogenously derived physiological regulator, based on its ability to affect intracellular signaling pathways [23]. CO has also emerged as a candidate experimental therapeutic molecule, based on its ability to dampen inflammatory responses in the context of in vitro and in vivo models of organ injury and disease [11,24]. The latter include acute kidney or lung injuries (AKI/ALI) and various forms of organ ischemia/reperfusion injury (IRI), which, in the context of organ transplantation, can predispose transplanted organs to dysfunction and acute or chronic graft rejection [11,25,26,27,28].

This review will examine the regulation and function of HO-1 in select human diseases where inflammation plays a key role in pathogenesis. Among these will include diseases of the lung [11], kidney [29,30] and CVD [31], and related metabolic diseases [32,33]. HO-1 has also been implicated in protective responses in other peripheral tissues such as bone and skin [34,35]. The controversial roles of HO-1 in neurodegenerative diseases [36] and cancer [37,38,39,40], which involve distinct mechanistic considerations, have been recently reviewed elsewhere.

### 1.1. Enzymology of Heme Degradation

**Figure 1:**
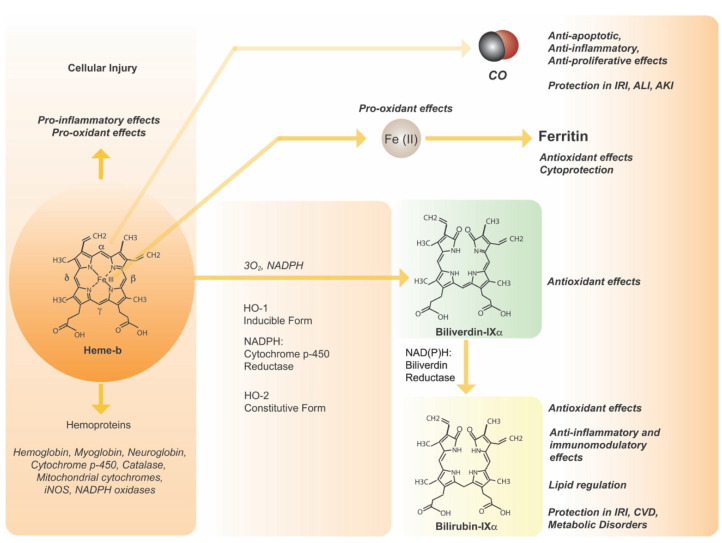
Enzymatic heme catabolism. Heme is a vital molecule used as a prosthetic group for diverse cellular heme-containing proteins, including oxygen carriers (i.e., hemoglobin, myoglobin), mitochondrial cytochromes, and enzymes. In free form, heme can catalyze pro-oxidant reactions and promote inflammation. Heme oxygenase (HO, E.C. 1:14:14:18) catalyzes the oxidative degradation of heme to biliverdin-IXα (BV). This reaction requires 3 mol O_2_ and NADPH: cytochrome p-450 reductase as the electron source. During heme cleavage, the α-methene bridge carbon of heme is released as carbon monoxide (CO), while the central heme iron is liberated as ferrous iron (Fe-II). HO activity is represented by an inducible form (HO-1) and a constitutively expressed form (HO-2). BV is converted to bilirubin-IXα (BR) by NAD(P)H: biliverdin reductase. Both BV and BR have potent cellular antioxidant activities. Additionally, BR has immunomodulatory effects, and a newly recognized role as a regulator of lipid metabolism. Iron released from HO activity may act as a pro-oxidant or trigger the synthesis of ferritin, which can act as a co-cytoprotectant by sequestering heme-derived iron. HO-1-derived CO can regulate cellular processes including inflammation, apoptosis, and cell proliferation. CO and BV/BR can confer protection in experimental models of ALI, AKI, CVD and related metabolic disorders, and organ IRI. Ferritin has also been implicated in vascular protection.

Heme oxygenase activity (HO; EC: 1:14:14:18) catalyzes the rate-determining step in oxidative heme degradation. HO activity generates one mol of BV per heme oxidized, with release of the heme iron, and evolution of CO as the product of release of the α-methene bridge carbon of heme (Figure 1) [7]. HO is not a hemoprotein at basal state but assumes transient hemoprotein characteristics upon heme (substrate) binding with a hexacoordinate configuration resembling that of myoglobin [41]. The HO enzymic reaction requires molecular oxygen (O_2_) and electrons donated from NADPH cytochrome p-450 reductase [7]. The ferrous heme iron is utilized for binding and activation of O_2_ which in turn executes subsequent autocatalytic cleavage of the heme molecule [7]. HO activity is represented by two major isoforms, an inducible isozyme (HO-1) and a constitutively expressed isozyme (HO-2), which are encoded by two distinct genes (*HMOX1* and *HMOX2*, respectively) [42]. HO-1 and HO-2 differ in biochemical properties including thermostability, *K_m_* for enzyme activity, and primary sequence, with ~43% amino acid sequence homology [43] Both proteins share a conserved 24-amino acid homologous region that defines the heme binding site, with a conserved histidine residue in this domain acting as axial heme iron ligand [42].

Both HO-1 and HO-2 also bear a conserved carboxyl-terminal region that serves as a membrane binding motif, that localizes the respective protein to the ER membrane [44,45]. In addition to the active site, HO-2 contains additional heme binding motifs that are not found in HO-1, designated heme regulatory motifs (HRM) [46,47,48]. These domains contain Cys-Pro motifs whose binding affinity for heme is subject to redox regulation. Ferric heme bound to HRMs may be reversibly transferred to the HO-2 catalytic site for degradation or released, dependent on redox status [49,50]. Binding of heme to HRMs of HO-2 also regulates the post-translational stability of HO-2 [51].

Apart from ER localization of HO-1, the subcellular redistribution of HO-1, under various stress-induced conditions, to various compartments, including the plasma membrane caveolae, mitochondria, and cell nucleus has also been described [52,53,54]. The significance of mitochondrial and caveolae localization of HO-1 remains incompletely delineated. HO-1 may regulate heme bioavailability in these compartments as well as provide a localized source of CO.

Caveolin-1 binding to HO-1 can inhibit HO-1 activity at the plasma membrane [53]. Consistently, caveolin-1 scaffolding domain peptides can promote anti-inflammatory effects by preventing the HO-1/caveolin-1 intermolecular interaction [55]. Furthermore, HO-1-derived CO was found to exert anti-inflammatory effects via promoting caveolin-1 interactions with the Toll-like receptor-4 (TLR4) [56].

The heme catabolic pathway is completed by BVR (E.C. 1.3.1.24) which reduces BV formed in the HO reaction to the lipophilic pigment BR [8]. BVR isolated from rat liver has pH-dependent dual specificity for both NADH and NADPH [57]. BVR, which exists in two isozymes (BVR-A, BVR-B) was originally characterized as a cytosolic enzyme, but its localization to other subcellular compartments, including caveolae and nucleus, have been described [53,58]. The eNOS-dependent nitrosylation of BVR was required for its nuclear translocation, and participation in an anti-inflammatory mechanism dependent on downregulation of TLR4 expression [58]. BVR is characterized as a Tyr/Ser/Thr kinase and interacts with other signal transduction proteins including insulin receptor kinase, protein kinase C isoforms, mitogen-activated protein kinases (MAPKs), phosphatidylinositol-3 kinase (PI3K)/Akt, and glycogen synthase kinase 3β (GSK3β) [59,60,61,62]. BVR-A has newly recognized roles in hepatic lipid regulation, as discussed below in the context of metabolic diseases, via regulation of a peroxisome proliferator activator-alpha (PPARα)-dependent pathway [62,63,64]. BVR can activate PPARα via phosphorylation-dependent inhibition of GSK3β, which relieves inhibition of PPARα, or directly via generation of BR, which binds to and activates PPARα.

### 1.2. Mechanisms of HO-Dependent Cytoprotection

Heme is naturally utilized as the prosthetic group for a variety of vital cellular enzymes and mitochondrial cytochromes [65,66]. Under normal physiological conditions heme serves as the cofactor for the principal oxygen (O_2_) carriers of the systemic circulation, muscle, and brain (i.e., hemoglobin, myoglobin, neuroglobin, respectively) [67]. Heme contains an iron center that participates in electron transfer reactions used in enzyme catalysis (e.g., cytochrome p-450-dependent drug metabolism). When heme is released into the circulation via intravascular hemolysis of red blood cells, or in the cellular milieu by oxidative modification of hemoproteins, heme can act as a potent pro-oxidant molecule, with potential to catalyze iron-dependent free-radical reactions. Heme can catalyze membrane lipid peroxidation and oxidize low density lipoprotein, with the potential to cause endothelial cell injury and promote atherogenesis [68,69,70]. Heme can also promote inflammatory responses via TLR4 [14]. Notably, iron released from HO-1 activity contributes to a transient labile iron pool that can catalyze adverse reactions, including peroxidation of membrane lipids and Fenton catalysis. Labile iron participates in the regulation of ferritin, which in turn sequesters the iron in a catalytically inactive form, and thereby acts as a detoxification mechanism and tissue cytoprotectant [71,72,73,74].

Studies of human HO-1 genetic deficiency have emphasized an important basal systemic role for HO-1 in organ homeostasis. The first reported HO-1 deficiency in humans presented with extensive endothelial cell damage, anemia, and aberrant iron deposition in tissues [75,76]. An additional eight reported cases of humans with nonsense or missense *Hmox1* mutations displayed similar pro-inflammatory phenotypes [76]. Seminal studies of HO-1 deficient mice (*Hmox1*^−/−^) have revealed fundamental roles for HO-1 in tissue iron homeostasis, protection from oxidative stress, and macrophage function [76,77,78,79]. Thus, the removal of heme and redistribution of heme iron remains one of the principal mechanisms by which HO can confer cytoprotection, especially under conditions where heme contributes to the pathogenesis.

The generation of other HO activity end-products, notably BV and CO, may individually or collectively contribute to the cytoprotective actions of HO-1. For example, BV and its conversion product BR, act as potent cellular and serum antioxidants [19,20]. More recent studies have identified a role for BR as a paracrine regulator of hepatic lipid content, by binding and activating PPARα. Application of bile pigments have therapeutic potential in various experimental models. BV has anti-inflammatory effects when applied to recipients or organ preservation fluid in models of transplant-associated IRI [80,81,82]. Hyperbilirubinemic states were shown to protect against experimental transplant-associated cardiac and hepatic IRI [83,84].

Importantly, CO has emerged as a potent endogenously produced physiological modulator due to its ability to affect intracellular signaling pathways. Among the known regulatory targets of CO include mitochondrial cytochromes and consequent modulation of mitochondrial redox homeostasis, which may trigger adaptive gene expression [23,24,85]. The classical proposed target of CO is the soluble guanylate cyclase sGC), which generates cyclic 3′–5′ guanosine monophosphate (cGMP) upon gaseous ligand binding, albeit with a weaker binding affinity than that of NO [86]. Additional signaling pathways that are modulated downstream by CO, as deduced by cell culture and animal model studies, include p38 MAPK, extracellular regulated kinase-1/-2 (ERK1/2) MAPK, c-Jun NH_2_ terminal kinase (JNK), signal transduction and activator of transcription-3 (STAT-3), phosphatidylinositol-3-kinase/Akt (PI3K/Akt), and hypoxia-inducible factor-1 (HIF-1) [23]. Exogenously applied CO can exert anti-apoptotic, anti-inflammatory, and anti-proliferative effects which collectively provide components of tissue protection [23]. Emerging evidence suggests that HO-1 or CO can exert immunomodulatory effects in various models, including transplant associated IRI [87]. Among these include the inhibition of T cell proliferation, B-cell maturation, and natural killer cell functions [87].

Non-canonical roles of HO-1 in cytoprotection, independent of HO enzymic activity or reaction products, have also been proposed [88,89]. These are exemplified in part by compartment-specific effects of a truncated form of HO-1 protein in the nucleus, and its potential protein-protein interactions, resulting in altered nuclear transcription factor activities [89,90]. HO-1 can also regulate heme bioavailability for G4 quadruplex formation, which are heme-dependent helical superstructures within chromatin, with novel implications for nuclear regulation and cancer cell proliferation [40].

### 1.3. HO-1 Transcriptional Regulation by Pro-Oxidants and Electrophilic Antioxidants

The upregulation of HO-1 responds to a broad spectrum of inducing stimuli, which include physical factors such as hemodynamic stress, thermal stress, or ultraviolet-A radiation exposure [91,92]. HO-1 induction also responds to phytochemical antioxidants, oxidative and nitrosative stress agents, dynamic changes in O_2_ tension, and exposure to chemical toxins such as heavy metals and thiol reactive substances [91,92]. The *Hmox1* gene (*Mus. musculus*) contains enhancer regions located at −4 kb and −10 kb relative to the transcriptional start site [93,94]. Human *HMOX1* promoter sequences bear similar but not identical upstream enhancer regions important for transcriptional responses [95,96]. Further, the proximal promoter regions of human, rat, and mouse *HMOX1* or *Hmox1* genes, respectively, contain additional *cis*-regulatory elements that may contribute to gene regulation in a context-specific fashion [93,97,98,99].

**Figure 2:**
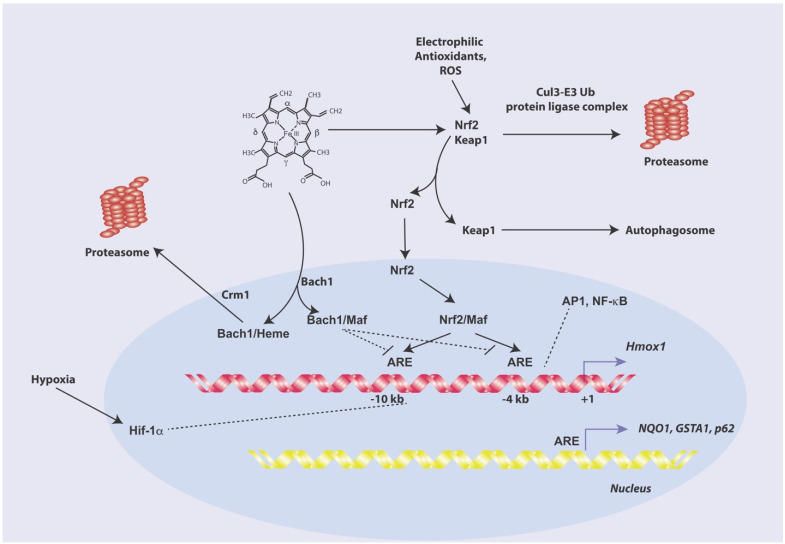
Regulation of HO-1 gene expression. The *Hmox1* gene contains distal enhancer regions located at −4 kb and −10 kb and additional *cis*-elements in the proximal promoter region. HO-1 responds to transcriptional regulation by heme, electrophilic compounds, ROS, heavy metals and other diverse stimuli. HO-1 gene expression in response to diverse agents is regulated by the nuclear factor erythroid 2–related factor 2 (Nrf2). Nrf2 forms stable heterodimers with small Maf proteins, which target antioxidant response elements (ARE) within the enhancers. The Kelch-like ECH-associated protein (Keap1), serves as a cytoplasmic anchor for Nrf2 under basal conditions, and facilitates its proteasomal degradation. When cells are exposed to inducing stimuli, Keap1 disengages from Nrf2, enabling Nrf2 to translocate to the nucleus where it can transactivate *Hmox1* gene expression. Nrf2 can also regulate other targets such as NAD(P)H: Quinone oxidoreductase 1 (*NQO1*) and glutathione S-transferase (*GSTA1*), and the autophagy substrate protein p62*^SQSTM1^*. The BTB and CNC homology-1 (Bach-1) protein acts as a transcriptional repressor of HO-1 and other Nrf2 target genes. Bach1 heterodimerizes with small Maf proteins to compete with Nrf2 for binding to ARE sequences in target gene promoters. Heme binding to Bach-1 inhibits DNA binding activity and promotes its nuclear export via the Crm1 transporter, leading to its proteasomal degradation. Additional transcription factors implicated in HO-1 transcriptional regulation include activator protein (AP)-1, hypoxia-inducible factor-1 (HIF-1), and nuclear factor-kappa-B (NF-κB).

The regulation of HO-1 gene expression in response to diverse stress agents is mainly operated by the nuclear factor erythroid 2–related factor 2 (Nrf2) acting as a *trans* factor [100] (Figure 2). A member of the Cap′n′collar/basic-leucine zipper family of proteins, Nrf2 can form stable heterodimers with small Maf proteins [100]. Nrf2 targets antioxidant response elements (ARE) intrinsic to the enhancer regions [101]. The Kelch-like ECH-associated protein (Keap1), which associates with the actin cytoskeleton, serves as a cytoplasmic anchor for Nrf2 under non-induced conditions, thereby inhibiting HO-1 expression [102,103]. The homodimerization of Keap1 via BTB/POZ domains is essential for its ability to sequester Nrf2 [103]. Keap1 binding enables the targeting of Nrf2 by the Cullin 3-based E3 ubiquitin ligase complex, which ubiquitinates Nrf2 for proteasomal degradation [104,105]. When cells are exposed to inducing stimuli, such as electrophilic antioxidants or CdCl_2_, Keap1 dissociates from Nrf2, thus enabling Nrf2 to translocate to the nucleus, where it can transactivate *Hmox1* gene expression [100,106,107].

In addition to HO-1 regulation, Nrf2 has been implicated in the regulation of Phase II proteins involved in cellular detoxification, such as NAD(P)H: Quinone oxidoreductase 1 (*NQO1*) and glutathione S-transferase (*GSTA1*). Nrf2 regulates the expression of the autophagy cargo adaptor protein p62*^SQSTM1^* which participates in the recruitment of substrates for autophagosome-lysosomal degradation [108]. p62*^SQSTM1^* reciprocally activates Nrf2 via binding and inhibition of Keap1 [109]. Nrf2 also promotes mitochondrial biogenesis via trans regulation of nuclear regulatory factor-1 and peroxisome proliferator-activated receptor gamma coactivator 1-alpha (PGC-1α) [110,111].

The BTB and CNC homology-1 (Bach-1) protein functions as a transcriptional repressor of HO-1 and other Nrf2 target genes. In addition to inhibiting *Hmox1* expression, emerging studies suggest that Bach-1 plays a complex role in tumor progression by regulating epithelial-mesenchymal transition, proliferation, angiogenesis, as well as sensitivity to cell death programs such as ferroptosis [112]. Bach1 forms stable heterodimers with small Maf proteins that compete with Nrf2 for binding to ARE sequences in target gene promoters [113,114,115]. Heme can inhibit the DNA binding activity of Bach-1 by direct binding to multiple Cys-Pro heme binding motifs [114,115,116]. Heme binding to Bach-1 also promotes its nuclear export via the Crm1 transporter, as well as its proteasomal degradation [117]. In addition to antagonizing Bach-1, heme inhibits the degradation of Nrf2 via the proteasome, thereby increasing HO-1 expression [118,119]. Several additional transcription factors have been implicated in HO-1 transcriptional regulation whose relative importance may vary with respect to model system as reviewed elsewhere [99,101].

### 1.4. Hmox1 Promoter Polymorphisms and Association with Human Disease

Non-coding polymorphisms can occur in the human *HMOX1* gene which impact gene regulation in carriers, and consequently these have been found to occur in association with many human diseases [120]. Microsatellite (GT)_n_ dinucleotide length polymorphisms were discovered in the promoter region of the human *HMOX1* gene, which can inhibit transcriptional regulation of *HMOX1* in carriers of the long (L) allele [i.e., (GT)_n_ ≥ 30] [121]. The L allele has been associated with susceptibility or severity to CVD, including coronary artery disease (CAD) and atherosclerosis [122,123,124]. Subjects homozygous for (GT)_n_ ≥ 32 had greater CVD risk, enhanced atherosclerosis progression, and displayed increased oxidative stress biomarkers [122]. CAD patients with reduced ejection fraction were found to have longer *HMOX1* promoter (GT)_n_ repeats than subjects with mid-range ejection fraction. The presence of _L_-allele was a predictor for diagnosis of low ejection fraction in CAD [123]. Shorter (GT)_n_ repeats in the *HMOX1* gene promoter were associated with increased survival in CAD patients with abnormal ejection fraction [124].

*HMOX1* (GT)_n_ ≥ 30 alleles have recently been described in association with severity of acute respiratory distress syndrome (ARDS) [125], and with potential significance in the outcome of SARS-CoV2 infection [126,127]. Additional associations were reported for sepsis-induced AKI [128], preeclampsia [129], and risk of Type 2 diabetes [130].

In chronic lung disease, L alleles of the (GT)_n_ repeat (variable lengths but typically ≥30) were correlated with emphysema [121], COPD susceptibility [131,132], and COPD severity [133], with response to therapy with *N*-acetyl-cysteine [134], lung function decline in COPD [135], and lung function decline in heavy smokers [136]. However, some studies failed to find association of *HMOX1* polymorphisms with lung function decline in smokers [137] or reported differential association of (GT)_n_ = 30, but not (GT)_n_ = 31 [138].

The *HMOX1* promoter gene also may bear a single-nucleotide polymorphism (SNP) rs2071746 (-413A>T) that has been analyzed for association with numerous disease conditions [120]. Recent studies indicate that A/A and A/T bone marrow (BM) donors were associated with a higher survival rate than T/T donors in bone marrow transplant recipients [139]. Taken together, these studies suggest that genetic variants in *HMOX1* gene promoter regions that inhibit gene expression may arise in subpopulations and may be linked to increased susceptibility to oxidative stress and related diseases. Additional studies will be necessary to confirm these associations in a disease-specific manner.

## 2. HO-1/CO in the Pathogenesis of Human Disease

**Figure 3:**
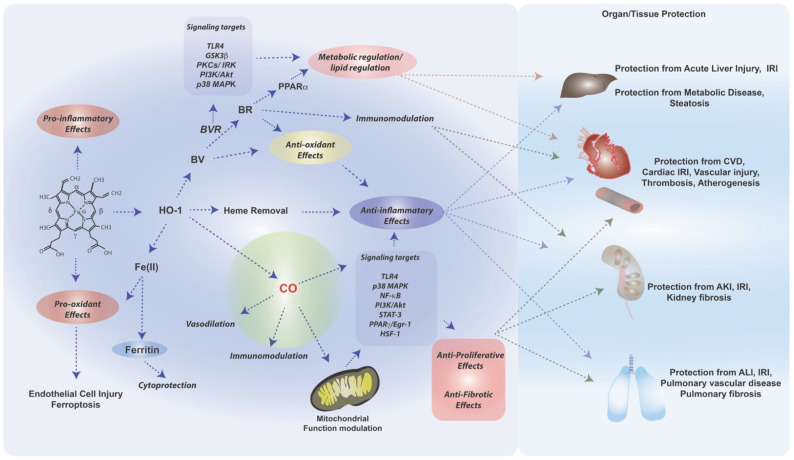
Role of HO-1/CO system in lung, vascular and kidney diseases. HO serves primarily a detoxification role in the removal of heme, which in free form can propagate inflammatory injury. Displacement of heme in exchange for labile iron may have toxic sequelae such as promotion of membrane lipid peroxidation and ferroptotic cell death. Labile iron triggers ferritin synthesis, which can contribute to vascular protection in CVD. Experimental models have suggested that BV/BR generation may contribute to protection in vascular injury, IRI, AKI, and ALI. BV and BR are known antioxidants. BR has a newly defined role as a regulator of lipid metabolism via activation of PPARα. BVR, which converts BV to BR, can modulate signaling pathways via intrinsic kinase activity. These effects may be important for regulation of lipid levels in the pathogenesis of liver and metabolic disorders. HO-derived CO generation (or exogenous CO application) triggers complex downstream signaling events with mitochondria believed to represent the proximal target. Among these include p38 MAPK and various other known signaling intermediates. CO-dependent signaling can trigger anti-inflammatory processes such as downregulation of neutrophil migration and macrophage pro-inflammatory cytokines production, that may provide protection in diverse conditions including vascular injury, liver injury, AKI, and ALI, and IRI in general. Additionally, CO can modulate smooth muscle proliferation, which can contribute to vascular protection. CO can also modulate fibroblast proliferation/activation, which may confer protection in organ fibrosis.

A wealth of information has been generated on the role of HO-1 and its reaction products in modulating the pathogenesis of select human diseases (Figure 3). The following sections will summarize the major studies in support of a protective role of the HO-1/CO system in models of acute and chronic disease, with an emphasis on cardiovascular, metabolic, lung and kidney diseases.

### 2.1. Protective Effect of HO-1 in Cardiovascular Disease (CVD)

Cardiovascular disease (CVD) remains the leading cause of death in developed nations, with ischemic heart disease the most prevalent [140]. CVD includes a range of conditions such as coronary artery disease (CAD), peripheral artery disease, cerebrovascular disease, and aortic atherosclerosis [141]. HO-1 and its reaction products can potentially impact and modify the pathogenesis of a wide variety of CVD-related conditions, including vascular injury, inflammation, and atherogenesis [9,31,142,143,144,145]. In early studies, HO-1 was found to inhibit vasoconstriction and cell proliferation during vascular injury [146]. Expression of HO-1 in arteries stimulated NO-independent sGC/cGMP-dependent vascular relaxation, and impaired vascular smooth muscle cell (VSMC) proliferation via upregulation of the cell-cycle inhibitor p21^Waf1/Cip1^. Expression of HO-1 inhibited lesion formation in pig arteries following vascular injury [146]. Induction of HO-1 by pre- and post-conditioning with hemin reduced vascular injury and neointimal hyperplasia after left carotid-artery balloon injury in rats, whereas co-treatment with the HO inhibitor SnPPIX reversed this protection [147].

Much insight into the role of HO-1 in various forms of CVD has also been generated from studies of HO-1 deficient mice. *Hmox1^−^*^/*−*^ mice were sensitized to arterial thrombosis and displayed hyperplastic arteries in response to vascular injury [146,148]. Endothelial cells (ECs) from *Hmox1^−^*^/*−*^ mice displayed increased apoptosis and denudation, and these mice also displayed increased von Willebrand Factor (vWF), tissue factor and reactive oxygen species (ROS) production, consistent with endothelial injury. Bone marrow (BM) transplantation from *Hmox1^−^*^/*−*^ donors conferred susceptibility to arterial thrombosis in response to vascular injury to recipient wild type mice. Furthermore, HO-1 was essential for regulating genes involved in cell cycle regulation, coagulation, thrombosis, and redox balance. Application of HO activity end-products, CO or BR, ameliorated the pro-thrombotic phenotype of *Hmox1^−^*^/*−*^ mice [148]. *Hmox1^−^*^/*−*^ mice were sensitized to tissue injury in a murine hindlimb ischemia model. Tissue protection associated with HO-1 was attributed to downstream modulation of Hif-1α, which coordinates adaptive responses to hypoxia. CO application compensated for HO-1 deficiency in this model [149].

Pre-exposure of rats or mice to CO (250 ppm) prevented atherosclerotic lesions after balloon injury or vascular transplantation [150]. These effects were related to inhibition of leukocyte infiltration in the graft. CO also exerted antiproliferative effects on vascular smooth muscle that were related to activation of sGC/cGMP, p38 MAPK, and increased expression of the cell cycle inhibitor p21^Waf1/Cip1^ [150]. The anti-proliferative effect of nitric oxide (NO) on VSMC in the rat balloon injury model required HO-1. However, the removal of HO-1 increased the pro-apoptotic effect of NO on VSMC in this model [151].

*Hmox1^−^*^/*−*^ mice were prone to atherosclerosis relative to their wild type counterparts [152]. Aged *Hmox1^−^*^/*−*^ mice displayed aortitis with monocyte infiltration and fatty streak formation, as well as lower body weight and elevated plasma triglycerides and lipid hydroperoxides. The ratio of apolipoprotein AI to AII in *Hmox1^−^*^/*−*^ mice was reduced in *Hmox1^−^*^/*−*^ mice relative to wild-type mice. Thus, HO-1 can suppress systemic inflammation in the artery wall and prevent plasma lipid peroxidation [152]. A possible pro-pathogenic role for HO-1-derived iron was implicated in diabetic vascular atherosclerosis. *Hmox1* deletion was found to reduce iron-dependent ferroptosis in this model [153].

HO-1 is implicated in organ protection in various cardiac injury models. For example, cardiac-specific transgenic mice overexpressing HO-1 had reduced infarct size, oxidative damage and inflammatory cell influx relative to wild type mice after cardiac IRI [154]. Vector driven HO-1 overexpression in rats also reduced cardiac fibrosis and remodeling in response to myocardial IRI [155].

In mice with sickle cell disease (SCD), HO-1 gene transfer inhibited hypoxia/reoxygenation-induced stasis. The increase of HO-1 expression in this model promoted anti-inflammatory effects associated with the activation (phosphorylation) of p38 MAPK and Akt, decreased the nuclear accumulation of p65 NF-κB in liver, and reduced circulating vascular cell adhesion molecule-1 (sVCAM-1) [156]. Pharmacological application of heme to increase HO-1 expression, inhibited vascular stasis and leukocyte adhesion, and inhibited NF-κB activation in lung and liver. Application of either CO by inhalation or BV also conferred anti-stasis and anti-inflammatory effects in this model [157,158]. Exposure to inhaled CO prevented hepatic necrosis, reduced BM myeloid and lymphoid markers, and reduced neutrophil and leukocyte counts in SCD mice [158]. A pro-pathogenic role for HO-1-derived iron was implicated in SCD. Towne ScD mice displayed higher levels of circulating heme and increase in cardiac ferroptosis markers. Application of the heme scavenging protein hemopexin or *Hmox1* deletion were found to reduce iron-dependent ferroptosis in this model and prevent cardiotoxicity/cardiomyopathy in SCD mice [17]. In contrast, HO-1 expression was found to aggravate doxorubin-dependent cardiomyopathy by release of excess heme-derived iron. This toxicity could be mitigated by metalloporphyrin inhibitors of HO activity (i.e., ZnPPIX), mitochondrial-targeted antioxidants, and metal chelators, and was reduced in Nrf2 deficient mice [159].

HO-1 has also been demonstrated to regulate blood pressure and confer vascular protection in experimental models of hypertension [160,161]. Endothelial targeting of HO-1 in angiotensin-II (Ang II) infused rats improved vascular function, reduced blood pressure, and reduced ROS and pro-inflammatory cytokines levels [161].

Cardiac overexpression of HO-1 in spontaneous hypertensive rats ameliorated hypertension in this model [162]. Retroviral-dependent HO-1 overexpression also reduced the pressor response to Ang II infusion in rats, the effects which were reversed by the HO inhibitor SnPPIX [163]. In *Hmox1^−^*^/*−*^ mice and their heterozygotes, the expression of NADPH: oxidase-2 (NOX2) was elevated in aortas relative to wild type mice. These mice were susceptible to Ang II infusion and expressed evidence of increased vascular dysfunction relative to wild type mice [164]. *Hmox1^−^*^/*−*^ mice displayed increased expression of chemokine receptor CCR2 in monocytes and aorta. Ang II-infused *Hmox1^−^*^/*−*^ mice presented with increased endothelial inflammation and increased aortic infiltration of pro-inflammatory CD11b^+^ Ly6C^hi^ monocytes and Ly6G^+^ neutrophils, relative to wild type mice. Furthermore, *Hmox1^−^*^/*−*^ mice exhibited Ly6C^hi^ monocytosis in the circulation and increased blood pressure response [164]. These studies concluded that HO-1 determines the phenotype of inflammatory and circulating infiltrating monocytes [164].

When subjected to Ang II-infusion, HO-1 deficient mice showed increased vascular dysfunction inversely correlated with HO activity [164]. In human studies of hypertensive individuals, monocyte HO-1 expression correlated with flow-mediated dilation and inversely with CD14 expression as an index of inflammatory monocytes [164].

Taken together, these studies suggest that HO-1 can exert anti-thrombotic effects and regulate anti-inflammatory and protective processes in vascular injury and disease. Potential adverse effects of HO-1 overexpression in these models may include the aggravation of tissue injury via iron-dependent ferroptosis. These observations underscore that therapeutic targeting of HO-1 expression must consider the balance of protective vs. detrimental effects in relation to the level of expression achieved.

### 2.2. Protective Effects of HO-1 and Its Reaction Products in Metabolic Diseases

Metabolic diseases such as obesity, metabolic syndrome, type 2 diabetes mellitus (T2DM), and hepatic disorders are significant contributors to worldwide morbidity and mortality and represent major underlying risk factors for CVD [165,166,167,168]. Obesity may lead to non-alcoholic fatty liver disease (NAFLD) which may progress to non-alcoholic steatohepatitis (NASH) or fatty liver. Chronic alcohol consumption may also promote steatosis. Hepatic steatosis in turn may progress to more serious conditions including hepatic fibrosis, cirrhosis, and hepatocellular carcinoma (HCC) [169,170].

HO-1, in conjunction with its enzymatic reaction products, has been implicated as a beneficial mediator across a spectrum various metabolic and/or hepatic diseases, by virtue of anti-inflammatory effects, as well as effects on adipocyte regulation, protection from lipotoxicity, and protection from cardiac remodeling [32,33,171,172].

Free heme causes hemolysis and can act as a catalyst of lipid peroxidation, leading to membrane damage and accumulation of lipid hydroperoxides, and may oxidize other targets, such as low-density lipoprotein (LDL); thereby contributing to endothelial dysfunction, loss of NO bioavailability, and leukocyte recruitment [70]. Thus, heme may contribute to the pathogenesis of metabolic disorders which typically involve inflammation, lipid accumulation and dysregulation. In this regard, the heme removal function of HO-1 may contribute to protection in metabolic diseases.

The metabolic protection associated with enhanced production of cardioprotective mediators, such as cytochrome P450 epoxygenase-derived epoxyeicosatrienoic (EET) acid and other lipid mediators, has been associated with HO-1 regulation [173]. EET supplementation ameliorated obesity-induced cardiomyopathy via upregulation of HO-1 and *Wnt* signaling [174]. In HFD fed *db/db* mice, therapy with EET agonist reduced FA accumulation, fibrosis, and NAFLD associated with increases in HO-1 and PGC-1α, improvement of mitochondrial functioning, and increases in insulin receptor phosphorylation [175].

Recent comparative studies of global KO mice (*Hmox1*, *Hmox2*) were performed to determine impact on basal metabolic functions [176]. Reduced insulin sensitivity and physical activity were observed in *Hmox1^−^*^/*−*^ but not *Hmox2^−^*^/*−*^ mice. *Hmox2* deletion increased proton leak and glycolysis in white adipose tissue (WAT) but not brown adipose tissue. The authors concluded that HO-1 is crucial for maintenance of insulin sensitivity, while HO-2 inhibits glycolysis and proton leak in the WAT under basal conditions [176].

Diabetic *Hmox1^−^*^/*−*^ mice were found more susceptible to myocardial IRI than their diabetic wild type counterparts and displayed left ventricular mural thrombi. Cardiac specific overexpression of HO-1 protected diabetic mice against myocardial IRI [177]. When subjected to streptozotocin-induced diabetes mellitus, HO-1 deficient mice showed increased vascular dysfunction inversely correlated with HO activity [164]. In a mouse model of methionine-choline-deficient (MCD) diet-induced NASH, administration of heme to induce HO-1 downregulated Wnt signaling and reduced hepatic steatosis, inflammation, fibrosis, and hepatic injury markers [178]. In human studies of subjects with NASH, HO-1 expression correlated with disease severity, ferritin levels, and degree of lipid peroxidation. HO-1 expression was inversely proportional to GSH levels. These studies provided early indication that HO-1 may represent a defense response in the context of clinical NASH [179].

Emerging studies have identified a cardinal role for BVR-A in mitigating susceptibility to metabolic disorders through activation of a PPARα-dependent pathway via regulation of glycogen synthase kinase-3β (GSK3β) [62,63,64]. Liver BVR-A was shown to protect against hepatic steatosis by inhibiting GSK3β via increasing inhibitory Ser-9 phosphorylation. GSK3β phosphorylates Ser-73 of PPARα, which inhibits activity and promotes proteolytic turnover. Liver-specific BVRA KO mice displayed increased GSK3β activity and decreased PPARα protein and activity. Liver-specific BVRA KO mice exhibited a phenotype of increased hepatic steatosis, increased plasma glucose and insulin levels and decreased glycogen storage. These authors identified a novel BVRA-GSKβ-PPARα pathway responsible for the regulation of hepatic lipid metabolism [62].

BR, generated by BVR, was shown to act independently as a paracrine regulator of lipid oxidation pathways by binding to and activating PPARα [63]. Bilirubin binding to PPARα results in inhibition of lipid accumulation. Bilirubin effects on lowering glucose and reducing body fat percentage were evident in wild type mice and these effects were abrogated in PPARα KO mice [63].

To further study the role of BVRA in fat regulation, a mouse model of adipose-specific deletion of biliverdin reductase-A (BVRA) (*Blvra*^FatKO^) was generated. In *Blvra*^FatKO^ mice subjected to high fat diet (HFD), these mice displayed higher visceral fat; and increased inflammation and decreases in mitochondrial number in WAT. *Blvra*^FatKO^ mice displayed decreased expression in the WAT of genes important in adipocyte regulation (i.e., *PPARα* and *Adrb3*). A phenotype of high fasting blood glucose levels without change in plasma insulin levels was also observed, associated with changes in WAT insulin signaling [180].

Recent studies using global BVR-A knockout mice (*Bvra*^−/−^) subjected to HFD lend further support to an essential systemic role for BVR in metabolic regulation. *Bvra*^−/−^ mice had normal insulin sensitivity and glucose metabolism relative to their wild type counterparts. These mice, however, displayed abnormal lipid accumulation, and increased oxidative stress markers, as well as depletion of the natural antioxidant α-tocopherol. The authors concluded that BVRA deficiency renders mice susceptible to oxidative stress-induced hepatic steatosis in the absence of insulin resistance [181,182]. In a mouse model of NAFLD, application of BR nanoparticles (pegylated BR) improved liver function and activated the hepatic β-oxidation pathway via PPARα [183]. Pegylated BR application also showed potential in mitigating hepatic IRI in mice [184].

In human studies, BVR-A levels were found to be significantly lower in patients with T2DM relative to non-T2DM subjects. Reduced BVR-A levels were associated with greater body mass, systolic blood pressure, fasting blood glucose, glycated hemoglobin, triglycerides, and pro-inflammatory and injury markers, and with lower high-density lipoprotein levels. Lower BVR-A levels were associated with reduced HO-1 protein levels. Low BVR-A levels were proposed as a predictor for T2DM [185]. Furthermore, reduced BVR-A expression in visceral adipose tissue of obese subjects was associated with development of NAFLD [186]. These studies underscore the significance of BVR-A in metabolic regulation.

Additional studies have shown the therapeutic potential of CO, when applied in gaseous form or as the product of carbon monoxide releasing molecules (CORMs) in metabolic disorders. In general, these effects are achieved by mitochondrial-specific effects, and also modulation of endoplasmic reticulum (ER) stress responses which contribute to metabolic phenotypes. For example, application of CORM-A1 (sodium boranocarbonate) prevented hepatic steatosis in a high fat high fructose diet-fed mouse model of NASH [187]. This effect was associated with improved lipid homeostasis, reduced oxidative stress, enhanced antioxidant responses, and improved mitochondrial function and respiration [187]. Oral administration of CORM-401 [Tetracarbonyl[*N-*(dithiocarboxy-?S,?S′)-*N*-methylglycine]manganate] to obese mice fed HFD improved body weight and glucose metabolism, and increased insulin sensitivity [188]. These effects were associated with CORM-401 localization to adipocytes, resulting in uncoupling of adipocyte mitochondrial respiration [188].

CO (applied as gas) or CORM-2 [Tricarbonyldichlororuthenium(II) dimer] conferred protection in an MCD-induced NAFLD model. The protection afforded by CO depended on mtROS-dependent and sestrin-1-dependent activation of the autophagy pathway. The effects of CO in this model were also dependent on upstream mediation by selective activation of the protein kinase R-like endoplasmic reticulum kinase (PERK), and downstream eIF2α–ATF4 signaling pathway, a branch of the unfolded protein response [189].

The protective effects of CO exposures against ER stress- or diet-induced, obesity-dependent hepatic steatosis were found to be dependent on increased fibroblast growth factor 21 (FGF21) expression in hepatocytes and liver. CO-stimulated PERK activation and enhanced the levels of FGF21 via the eIF2α–ATF4 signaling pathway. Inhaled CO lowered blood glucose levels, enhanced insulin sensitivity, and promoted energy expenditure in mice by stimulating browning of the WAT. These studies concluded that CO critically depends on FGF21 to regulate metabolic homeostasis [190].

In addition, the HO-1 system has been described as a protective mediator in various forms of acute liver injury, including IRI incurred by orthotropic liver transplantation. The protective effects of HO-1 and its reaction products in liver IRI involve stimulation of the homeostatic autophagy program via Sirt1 activation and related anti-inflammatory effects [191,192]. In human liver transplant biopsies, high HO-1 levels were correlated with well-preserved hepatocellular function and enhanced SIRT1 and autophagy markers [192].

CO application also exerts protective effects on hepatic IRI in various models [28]. Recent studies show that application of CO via CORM-2 provided protection against hepatic IRI in rats. In this model CO increased SIRT1 expression, which was found to deacetylate high mobility group box 1 (HMGB1) and subsequently reduce its translocation and release, as a mechanism for hepatoprotection [193]. In earlier studies of rat orthotropic liver transplantation, CO gas inhalation protected against transplant IRI but reducing pro-inflammatory mediators and neutrophil influx into the graft [194].

### 2.3. Protective Effect of HO-1 in Renal Diseases

HO-1 expression has been observed to confer protection in various models of acute kidney injury (AKI) in rodents via anti-inflammatory and/or immunomodulatory effects [87]. These studies include models of AKI induced by ischemia/reperfusion (I/R), sepsis, or chemotherapeutics [29,30]. The upregulation of HO-1 expression is well characterized as a stress response to IRI in the kidney [195]. The release of free heme, a pro-oxidant molecule, was associated with pathological responses to prolonged warm ischemia and reperfusion [195]. Increases in labile heme levels and HO-1 expression correlated with the elevation of inflammatory markers and complement factors during kidney IRI [195].

Recent genetic studies in mice revealed that myeloid-specific HO-1 knockout mice were susceptible to I/R-induced AKI with evidence of increased renal inflammation and apoptosis [196]. Specific overexpression of HO-1 in the thick ascending loop of Henle (TALH) attenuated Ang II-induced hypertension, by a mechanism that potentially involved the modulation in NKCC2-dependent sodium reabsorption [197]. Mitochondria-specific HO-1 expression conferred protection to renal proximal tubule epithelial cells against hypoxia-induced toxicity and oxidative stress [198].

Heme has been shown to be effective as a pre-conditioning agent to upregulate the cytoprotective HO response in kidney IRI [195]. HO-1 induced by heme pretreatment, provided protection against renal IRI by inhibiting pro-inflammatory cytokine responses. Heme preconditioning also protected the lung against ALI secondary to renal IRI, by reducing neutrophil influx and pro-inflammatory cytokines production [199]. The timing of heme administration to induce HO-1 was shown to be a critical variable, as application of heme after I/R surgery promoted IRI [200]. Preconditioning of wild type mice with heme prior to I/R surgery conferred protection and resulted in increased HO-1 expression specifically in CD11b^+^ F4/80^lo^ renal myeloid cells [196].

CO treatment alleviated transplant-associated IRI in a rat model of kidney transplantation, by downregulating the expression of pro-inflammatory mediators [81]. Application of BV was also effective in this regard, whereas combined treatments with both BV and CO were most effective at dampening oxidative stress and improving blood flow in the graft [81]. Application of CO to a pig model of cardiopulmonary bypass alleviated biochemical and histological markers of AKI, and this effect was related in part to upregulation of heat shock protein-70 (Hsp70) [201].

Iron-dependent ferroptosis has emerged as a contributor to the pathogenesis of AKI [202]. A relationship between HO-1 and ferroptosis has also been described in the context of AKI. *Db/db* mice displayed a phenotype of AKI with enhanced ferroptosis. These mice were protected from AKI by application of the ferroptosis inhibitor ferrostatin. The protection conferred by ferrostatin was associated with reduction of HO-1 levels in this model [203]. In vitro studies, however, using *Hmox1* deficient renal epithelial cells suggest a protective effect of HO-1 in limiting chemically induced ferroptosis [204]. Furthermore, recent studies identify a pathway by which inhibition of miR-3587 in renal epithelial cells results in increased HO-1 expression, which was associated with upregulation of glutathione peroxidase-4 and protection from ferroptosis in vitro [205].

Overexpression of HO-1 also prevented renal fibrosis during unilateral ureter obstruction (UUO). This effect was associated with reduction of inflammation and inhibition of myofibroblast activation and proliferation, and tubulointerstitial infiltration of macrophages. Further, the effects of HO-1 were related to prevention of Wnt/β-catenin signaling [206]. In the UUO model, exposure to CO reduced markers of kidney fibrosis, in a manner dependent on the MKK3 pathway [207].

Similar to effects observed in models of metabolic disease, BVR-A has a potential role in lipid regulation in the kidney. In a model of [clustered regularly interspaced short palindromic repeats (CRISPR)]-dependent knockdown of BVR-A in cultured renal proximal tubule cells, BVR-A deficient cells displayed reduced metabolic potential and mitochondrial respiration and increased intracellular accumulation of intracellular triglycerides [208]. These cells also displayed enhanced susceptibility to palmitate-induced lipotoxicity. These experiments underscore a potential role for BVR-A in mitigating lipotoxicity in the kidney in the context of kidney diseases [208].

### 2.4. Anti-Inflammatory Effects of HO-1 in Acute Lung Injury

Protective effects of the HO-1/CO system and related metabolites have been demonstrated in pre-clinical models of acute lung injury (ALI) [11]. Of these, the most prominent are models of hyperoxia, sepsis, and ventilator-induced lung injury (VILI). In rodent models of LPS-induced inflammatory lung injury, HO-1 expression by gene transfer protected against aerosolized LPS-induced ALI in mice via limiting neutrophil influx and pro-inflammatory responses. These effects were associated in part with the increased production of the anti-inflammatory cytokine IL-10 [209]. CO treatment (250 ppm) also protected against LPS-induced ALI associated with p38 MAPK-dependent downregulation of pro-inflammatory cytokines responses, and upregulation of IL-10 [210]. HO-1 expression by gene transfer also protected against ALI induced by Type A influenza virus infection. HO-1 reduced inflammatory cell influx and inhibited caspase-8 dependent apoptosis in this model [211].

Hyperoxia (>95% O_2_) is used clinically in the management of patients with acute, severe respiratory failure. In rodents, exposure to hyperoxia can cause ALI, associated with increased oxidative stress and lung inflammatory responses, and damage to respiratory endothelium and epithelium [212]. Rodents subjected to hyperoxia (>95% O_2_) develop inflammatory lung injury characterized by neutrophil influx in the airways, pulmonary edema, pleural effusion, and an increased pulmonary cell apoptosis. Gene transfer of *Hmox1* in rat lungs, which increased HO-1 expression in the bronchiolar epithelium, protected against the development of ALI and increased survival during hyperoxia exposure [213]. Anti-inflammatory effects were observed in mice treated with inhaled CO prior to hyperoxia exposure, a model of ALI [214,215]. Inhaled CO (250 ppm) during hyperoxia exposure prolonged the survival of rats and mice subjected to a lethal exposure to hyperoxia, and reduced histological indices of lung injury, including airway neutrophil infiltration, fibrin deposition, alveolar proteinosis, pulmonary edema, and apoptosis, relative to animals exposed to hyperoxia without CO [214,215]. In mice, hyperoxia was shown to induce the expression of pro-inflammatory cytokines (i.e., TNF-α, IL-1β, IL-6) and activate major MAPK pathways in lung tissue. The protection afforded by CO treatment against the lethal effects of hyperoxia correlated with the inhibited release of pro-inflammatory cytokines in the bronchioalveolar lavage. The protective effects of CO in the hyperoxia-induced ALI model required the MKK3/p38β MAPK pathway [216].

Mechanical ventilation (MV) can cause ALI, termed ventilator-induced lung injury (VILI) in rodents associated with inflammation and neutrophil migration into the airways. Rats ventilated with an injurious (high tidal volume) ventilator setting in the presence of intraperitoneal LPS exhibited increased expression of HO-1 in the lung [216]. MV at moderate tidal volume (12 mL/kg), without pro-inflammatory challenge, can also induce HO-1 protein expression in the mouse lung [217]. Inclusion of CO in the ventilator circuit (e.g., 250 ppm) conferred protection in rodent VILI models by a mechanism primarily involving inhibition of neutrophil influx [217,218].

ALI may lead to severe respiratory disease or acute respiratory disease syndrome (ARDS). HO-1 has also been studied in the context of clinical ARDS. HO-1 protein levels were elevated in lung tissue and in BAL fluid from patients with ARDS compared with control subjects. Levels of HO-1 protein in BAL fluid from patients with ARDS correlated with changes in the concentrations of ferritin and the iron saturation of transferrin but were inversely correlated with bleomycin-detectable iron [219]. The utility of serum HO-1 as a biomarker of ARDS was recently reevaluated [220]. Serum HO-1 level at diagnosis was significantly higher in ARDS patients than in patients with acute exacerbation of interstitial lung disease (AE-ILD) patients [220]. HO-1 expression was correlated with serum BR and LDH and generally declined two weeks post-diagnosis in patient both groups but remained elevated [220]. Serum HO-1 levels at the time of diagnosis combined with age, sex, and partial pressure of oxygen in arterial blood/fraction of inspired oxygen (P/F) ratio may be used to predict three-month mortality in ARDS patients [220].

### 2.5. Pulmonary Fibrosis

Idiopathic pulmonary fibrosis (IPF) is a common form of interstitial lung disease (ILD) characterized by scarring or thickening of lung tissues associated with fibroblast hyperproliferation and extracellular matrix (ECM) remodeling with unclear etiology and currently limited therapeutic options [221]. IPF affects primarily the lower respiratory tract resulting in compromised efficiency of alveolar gas exchange [221]. In mouse models of pulmonary fibrosis, HO-1 gene transfer provided protection against bleomycin (BLM)-induced fibrotic lung injury in mice [222]. Exogenous CO treatment by inhalation was also shown to provide protection against BLM-induced fibrosis in mice [223]. In this model, CO-treated animals displayed reduced lung hydroxyproline, collagen, and fibronectin levels relative to air-exposed BLM-injured controls. The protective effect of CO in the BLM-induced fibrosis model was associated with an anti-proliferative effect of CO on fibroblast proliferation involving the increased expression of p21*^Waf1/Cip1^* and inhibition of cyclins A/D expression [223]. Similar anti-fibrotic effects in BLM-induced pulmonary fibrosis were reported using application of a novel nanocarrier, CO-bound hemoglobin-vesicles (CO-HbV) [224].

HO-1 expression increases in lung tissues of patients with ILD, to signify anti-inflammatory M2 macrophage activation. Measurement of HO-1 levels in peripheral blood could provide a useful biomarker for the severity of lung injury in ILD and for predicting fibrosis generation [225]. As found for ARDS patients, serum HO-1 levels at the time of diagnosis combined with age, sex, and P/F ratio may also be useful to predict three-month mortality in ILD patients with acute exacerbations [220].

### 2.6. Cystic Fibrosis

Cystic fibrosis (CF) is an inherited disorder caused by mutations in the cystic fibrosis transmembrane conductance regulator gene (*CFTR*) [226]. The pathogenesis of CF also involves opportunistic Gram-positive *Staphylococcus aureus* and Gram-negative *Pseudomonas aeruginosa* (PA) infections [227]. Modulation of the HO-1/CO system has shown therapeutic potential in experimental models of CF [228]. Early studies have demonstrated elevated HO-1 and ferritin expression in macrophages of patients with CF [227]. HO-1 expression conferred protection against PA-induced apoptosis in airway epithelial cells [229,230]. In vivo studies also demonstrate reduction of neutrophil influx and protection from airway epithelial cell apoptosis in response to PA infection in mice [230].

Recent studies in CF patients uncover a miR-125b-dependent pathway for regulation of Nrf2 and HO-1 that coordinate protective responses. In nasal epithelial cells, the expression levels of HO-1 and miR-125*b* positively correlated with a forced expiratory volume in 1 s (FEV1) of greater than 60% in patients with CF with chronic PA lung infection [231]. Observed increases in the stability of Bach1 and elevated miR-155 expression in CF bronchial epithelial cells which may contribute to HO-1 deficiency in these cells [232]. CF airway epithelial cells also displayed increased ferrous iron accumulation, and reduced antioxidant defense, contributing to a phenotype of increased susceptibility to ferroptosis [233].

## 3. Conclusions/Therapeutic Considerations

The targeted upregulation of HO-1 may be considered as a candidate therapeutic strategy to mitigate inflammation or fibrosis in organ diseases. Modalities to achieve an elevated HO-1 expression may include global or tissue-specific vector-dependent overexpression approaches (i.e., gene therapy) or depend on chemical/pharmacological inducers of the Nrf2/HO-1 system. These may include the natural substrate heme and derivatives (e.g., CoPPIX), natural antioxidants such as curcumin [106,234], or synthetic Nrf2-targeting compounds such as dimethylfumarate and others [235]. For example, natural product inducers of HO-1 such as curcumin and resveratrol have shown potential as therapies for metabolic diseases [6,33]. In addition to serving as an effective pre-conditioning agent, heme may also contribute directly to the pathogenesis of inflammatory conditions. Therefore, strategies for therapeutic modulation of HO-1 must consider toxicity of inducing compounds, timing of inducer administration (pre- or post-injury), kinetics of HO-1 expression relative to injury, as well as degree of HO-1 expression. Thus, for therapeutic efficacy, HO-1 expression must be tightly regulated [236]. The pleiotropic effects of HO-1 activity and its end-products (CO, BV/BR, Fe II) imply that multiple biological sequelae may be involved, which may contribute to net beneficial effects or also to potential adverse effects at low or high HO-1 expression. These may include undesired contextual modulation of apoptosis or activation of other cell death pathways such as iron-dependent ferroptosis or pyroptosis [236]. In addition to production of its end-products, non-canonical effects of HO-1, such as modulation of nuclear transcription factor activities must also be considered when designing HO targeted approaches [89,90]. Strategies involving the inhibition of HO activity with metalloporphyrins and other synthetic inhibitor compounds will have similar considerations [237].

Therapeutic application of HO-end products, such as BV and BR have been described in models of organ transplant-induced IRI, AKI, and ALI. Emerging studies also suggest that the BVRα/GSKα/PPARα axis may provide an attractive therapeutic target in metabolic disease and related cardiovascular or renal conditions [22,64]. For example, BR nanoparticles were shown to be effective at improving liver function in NAFLD [183].

CO has demonstrated pre-clinical efficacy in model systems when applied exogenously. Many of these studies have applied CO in gaseous form to cells and animal model systems. For pharmacological delivery of CO in lieu of gas, a number of transition metal-based carbon monoxide releasing molecules (CORMs) have been developed, and these also have shown efficacy in animal models of inflammatory disease [238,239].

Therapies dependent on CO application must consider dose-dependent effects, route of administration, and mode of administration (i.e., in the case of CORMs and other CO-donor compounds, or inhalation gas). In the case of CORMs, potential toxicological effects of metal centers or CO-independent effects of the backbone must also be considered [27,240,241,242]. The current status of the clinical development for inhaled CO for various human indications, including applications in ARDS and pulmonary fibrosis [243,244], has been extensively reviewed elsewhere [245,246]. Further progress in this area will be contingent on the completion of pending or planned clinical trials.
